# Let's Make Those Book Chapters Open Too!

**DOI:** 10.1371/journal.pcbi.1002941

**Published:** 2013-02-21

**Authors:** Philip E. Bourne

**Affiliations:** 1 Department of Pharmacology, University of California San Diego, La Jolla, California, United States of America; 2 Skaggs School of Pharmacy and Pharmaceutical Sciences, University of California San Diego, La Jolla, California, United States of America

As authors, many of us have had less than satisfactory experiences in writing book chapters as part of a themed volume or textbook that, when published, are expensive, inaccessible, and cited and used infrequently through lack of availability [1]. It could even be argued we write them from some sense of obligation and need, but do not put our best science and efforts into them because we know they won't be read and hence cited. Putting that thought aside, let's just say that a lot of good science goes underutilized. Some finds its way into journal reviews and journals that specialize in such content, for example the Elsevier *Current Opinions* series, but much languishes. I, many of the PLOS editors, and PLOS management have long wanted this situation to change; well, now it has.

The value of themed hardcover volumes and textbooks was understandable in a purely print era, an era during which we frequented the library more, which is where these volumes resided, being too expensive for individuals to purchase. These volumes make no sense today in a digital, open-access world. We are proud to report that *PLOS Computational Biology* has taken the first steps to address this nonsense. *Translational Bioinformatics,* edited by Guest Editor Maricel Kann and Education Editor Fran Lewitter, is the first complete PLOS “book” that can be accessed online as individual chapters or downloaded as a complete volume [2]. An ePub version is now available too. The content is indexed, each chapter has a Digital Object Identifier (doi) assigned and hence is resolvable (i.e., uniquely findable), and is indexed in PubMed and available as full text from PubMed Central, like all PLOS content.

*Translational Bioinformatics* can be used as a reference guide or textbook, and includes exercises. After review, the authors have had their hard work rewarded through a PLOS citation and greater accessibility to their work. The book uses the PLOS collection feature to bring the content together into a single entity while retaining the individuality of each article. While we regard this as an important step forward, it does raise some questions.

The first question is, who pays? Obviously, we are strong proponents of open access, but also the first to admit there must be a business model if open-access content is to be persistent. Certainly, most of us have never made any money from writing specialized book chapters contributed to a volume, but would we pay a modest amount to have them published under an open–access license? This remains an open question at this time. PLOS met the cost of publishing *Translational Bioinformatics*, but if this approach to book chapters were to take off, someone will have to foot the bill. At this time, PLOS is interested in furthering this cause, and, as with all front matter within the Education Section of the journal, book chapters are not subject to publishing fees. If the demand becomes too great we will need to revisit this. Support for chapter content would seem an opportunity for a wonderful contribution by an individual philanthropist or foundation in furthering scientific dissemination.

The second question is, what quality of review do we require of chapter content? In general terms, solicited book chapters do not undergo the level of review found in a research article. Unless the content is terrible, the editors soliciting the material are hard pressed to reject it, having persuaded the authors to write it in the first place. Good editors, as we have here, will provide the level of review found in a research article. Moreover, with greater exposure and article-level metrics (ALMs) applied to each chapter, the content will rise or fall on its own merits and the end result will likely be higher quality content than we have traditionally seen from book chapters. With regard to *PLOS Computational Biology* specifically, we regard this book content as front matter in the Education Section, and as such it has had significant review. We will be revisiting the issue of review, and indeed all aspects of the scope of our support for chapters, as demand increases.

The third question is, what do we lose and gain in an online book? Of course there are the obvious issues, now long debated, regarding ebooks versus physical books, and there is no need to revisit that here. What is worth visiting are the specific issues surrounding a book publication by PLOS, an organization that is currently set up to operate as a journal publisher. PLOS has been wonderful in making this project happen and paving the way for more through the notion of collections. Collections do present challenges when used to represent a book. For example, the collection has no ISBN or other book-like identifier defining the citation; books have editions, whereas there is no notion of versioning in a collection. On the positive side, the collection can become a dynamic entity, such that new chapters (with their own journal-like citation) can be added to the collection at any time.

Open questions there might be, but an exciting time nevertheless, with PLOS continuing to push the envelope regarding scholarly communication. Over time we will sort this out, but in the meantime, enjoy *Translational Bioinformatics,* a new innovation in open-access publishing.
